# Macrocytic anemia is associated with the severity of liver impairment in patients with hepatitis B virus-related decompensated cirrhosis: a retrospective cross-sectional study

**DOI:** 10.1186/s12876-018-0893-9

**Published:** 2018-11-01

**Authors:** Jian Yang, Bin Yan, Lihong Yang, Huimin Li, Yajuan Fan, Feng Zhu, Jie Zheng, Xiancang Ma

**Affiliations:** 1 Clinical Research Center, the First Affiliated Hospital, Xi’an Jiaotong University, Xi’an, 710061 People’s Republic of China; 2 Department of Psychiatry, the First Affiliated Hospital, Xi’an Jiaotong University, No.277 Yanta West Road, Yanta District, Xi’an, 710061 People’s Republic of China; 3 Center for Translational Medicine, the First Affiliated Hospital, Xi’an Jiaotong University, Xi’an, 710061 People’s Republic of China

**Keywords:** Macrocytic anemia, HBV-related decompensated cirrhosis, MELD score, Severity of liver impairment

## Abstract

**Background:**

Macrocytic anemia is common in liver disease. However, its role in hepatitis B virus (HBV)-related decompensated cirrhosis remains unknown. The aim of the present study was to determine the association between macrocytic anemia and the severity of liver impairment in patients with HBV-related decompensated cirrhosis according to the Model for End Stage Liver Disease (MELD) score.

**Methods:**

A total of 463 participants who fulfilled our criteria were enrolled in this cross-sectional study. Patients were classified into three groups according to anemia types, diagnosed based on their mean corpuscular volume level. Multivariate linear regression analyses were used to determine the association between macrocytic anemia and the MELD score for patients with HBV-related decompensated cirrhosis.

**Results:**

Patients with macrocytic anemia had evidently higher MELD scores (10.8 ± 6.6) than those with normocytic anemia (8.0 ± 5.5) or microcytic anemia (6.3 ± 5.1). The association remained robust after adjusting for age, gender, smoking, drinking, and total cholesterol (β = 1.94, CI: 0.81–3.07, *P* < 0.001).

**Conclusions:**

Macrocytic anemia was found to be associated with the severity of liver impairment and might be a predictor for short-term mortality in patients with HBV-related decompensated cirrhosis.

## Background

Cirrhosis is an end-stage disease that invariably leads to death. It is the 14th most common cause of death in adults worldwide and results in 1.03 million deaths per year [1]. Chronic infection with hepatitis B virus (HBV) is one of the major causes of cirrhosis and 30% of deaths are attributable to HBV [2, 3]. China is a highly endemic area of HBV, where 78% of patients with cirrhosis are HBsAg positive [4]. In patients with cirrhosis, the 5-year probability of decompensation is 15–20%, while the 5-year survival rate decreases from 84 to 14–35% once clinical decompensating events occur [5–7].

Anemia is a common comorbidity in cirrhosis that is associated with poor prognosis [8]. Erythrocyte abnormalities were clinically important and frequent findings in patients with chronic disease. Mean corpuscular volume (MCV), a measurement of the average volume of red blood cells (RBCs), has been documented to be associated with an increase in many clinical conditions [9–12]. Typically, anemia can be classified into macrocytic anemia (> 100 fL), normocytic anemia (80–100 fL), and microcytic anemia (< 80 fL) based on the patient’s MCV level. A recent study has reported that the elevated MCV level was associated with increased liver cancer mortality, especially in men who are hepatitis B surface antigen (HBsAg) positive [13]. Therefore, in this study, we hypothesized that a common association might exist between macrocytic anemia and the severity of liver impairment in patients with HBV-related decompensated cirrhosis.

We used the Model for End Stage Liver Disease (MELD) score for evaluating the severity of liver impairment of HBV-related decompensated cirrhosis. The MELD score was developed to predict the short-term mortality of end-stage liver disease because of the shortage of donated livers. It had been validated subsequently as an accurate predictor of survival among different populations of patients with advanced liver disease and was adopted for organ allocation for liver transplantation instead of the older Child-Pugh score in the USA since 2002 [14–16]. Liver transplantation is generally recommended for patients with MELD score of > 15, if possible [17].

The goal of the present study is to investigate whether the MELD score is higher in the macrocytic anemia group in patients with HBV-related decompensated cirrhosis.

## Methods

### Study population

**Fig. 1:**
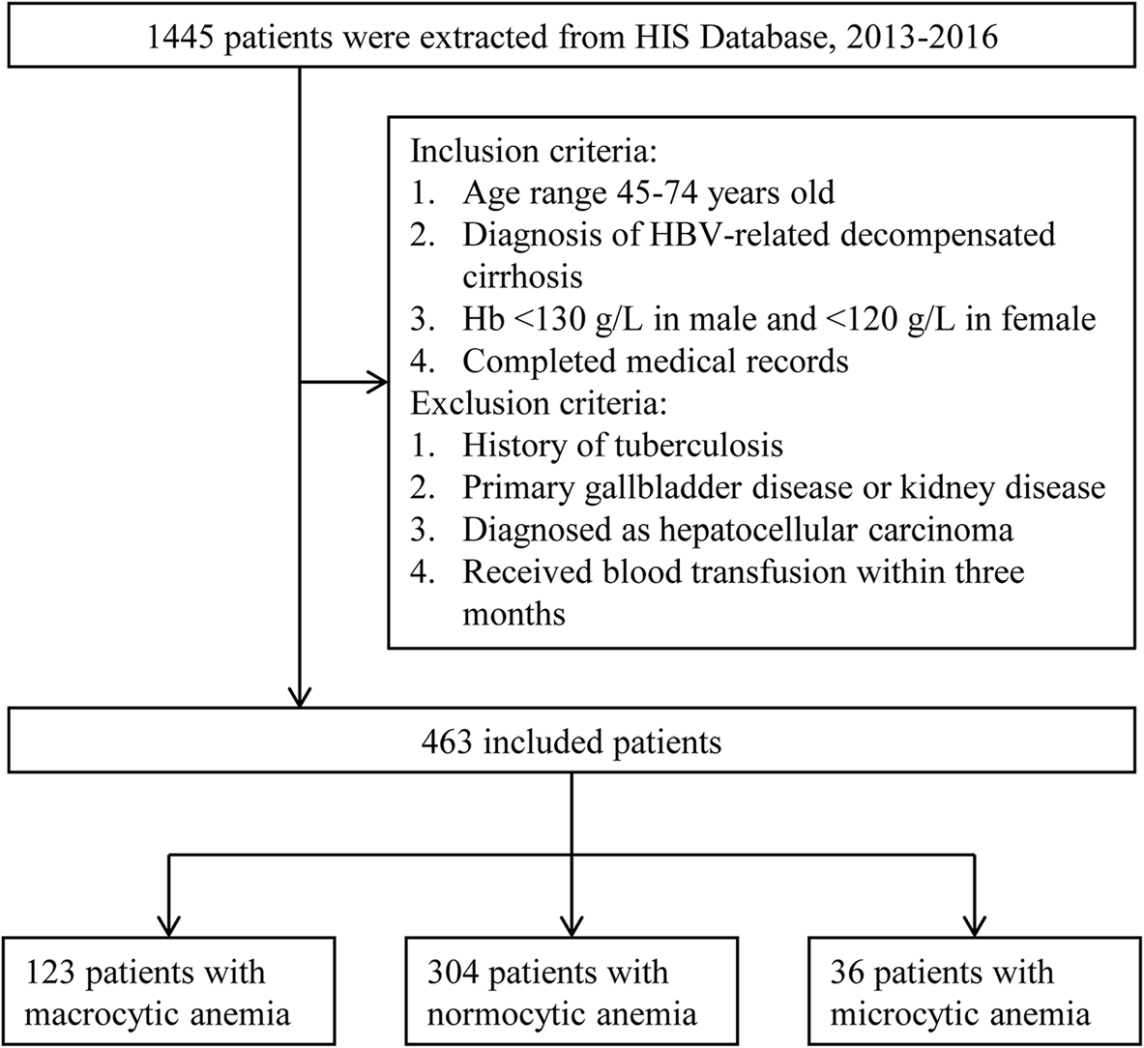
Flow diagram for the selection of patients

From May 2013 to July 2016, data of 1445 patients diagnosed as having HBV-related decompensated cirrhosis were extracted from the HIS Database at the First Affiliated Hospital of Xi’an Jiaotong University. For patients to be diagnosed as having HBV-related decompensated cirrhosis, the following conditions must be present: HBsAg carrier for ≥6 months; pathological or clinical evidence of cirrhosis; and occurrence of complications, such as ascites, upper gastrointestinal bleeding, spontaneous bacterial peritonitis, or hepatic encephalopathy [6, 18–20]. Anemia was defined according to WHO’s haemoglobin thresholds, which is haemoglobin level of < 130 g/L in male and < 120 g/L in female [8]. After strictly screening according to the inclusion criteria and exclusion criteria, 463 patients were enrolled in this hospital-based cross-sectional study (Fig. 1). The study was approved by the Ethics Committee of the First Affiliated Hospital, Xi’an Jiaotong University. Since this is a retrospective study, a written consent is waived by the Ethics Committee and is deemed unnecessary. All methods were carried out in accordance with appropriate clinical practice guidelines and national legal requirements.

### Data collection

Demographic characteristics were obtained from an interview during the patients’ admission to our hospital. Venous blood samples were collected from the participants after an overnight fasting for laboratory assessments. Smoking was defined as having ≥1 cigarette per day and drinking was defined as alcohol intaking > 20 g per day for at least a year [21, 22]. Estimated glomerular filtration rate (eGFR) was calculated using a formula adapted from the Modification of Diet in Renal Disease (MDRD) equation [23, 24]. Unfortunately, body mass index (BMI) and HBV DNA data were not included in the analysis due to excessive missing values.

### MELD score

The MELD score was calculated using the following formula: 9.57 × log_e_ (creatinine mg/dl) + 3.78 × log_e_ (bilirubin mg/dl) + 11.2 × log_e_ (INR) + 6.43, where INR is the international normalised ratio and 6.43 is the constant for liver disease aetiology [16].

### Statistical analysis

Statistical analyses were conducted using R software (version 3.1.3). Continuous data were presented as mean ± SD, and categorical variables were presented as count and percentage. All participants were divided into three groups according to their anemia classification. We used one-way ANOVA to determine the differences among the three groups in terms of the continuous variables, because the variables were all normally distributed and homogeneous in variance. Simultaneously, the chi-square test was used for categorical variables. Univariate and multivariate linear regression analyses were used to examine the associations of the MELD score with macrocytic anemia. Variables with *P* value < 0.05 in univariate models were then included in the multivariate analyses. A two-tailed test was used to calculate the P value, and the results were considered statistically significant when the *P* value < 0.05.

## Results

### Characteristics of participants

Table 1 presents the baseline characteristics of the participants, which were divided into three groups according to anemia types. Among the 463 eligible participants, 304 had normocytic anemia, 123 had macrocytic anemia and 36 had microcytic anemia. The average age of participants was 54.3 (SD = 7.3) years and 63.5% of them were male. Our data showed that patients with macrocytic anemia were older and had higher levels of bilirubin, international normalized ratio (INR) and alkaline phosphatase (ALP) compared to patients with normocytic or microcytic anemia. MELD score was also observed to be higher in the macrocytic group. Oppositely, the total cholesterol and albumin were relatively low. There were no significant differences observed in terms of gender, smoking, drinking, hypertension, systolic blood pressure, diastolic blood pressure, creatinine, eGFR, aspartate aminotransferase (AST) and alanine aminotransferase (ALT). The haemoglobin level and prevalence of diabetes in the microcytic group were slightly different from that in the other two groups, but this difference was negligible.

**Table 1:** Demographic and biochemical characteristics of the study participants (*N* = 463)

Variable	Macrocytic anemia	Normocytic anemia	Microcytic anemia	*P* value
Number of subjects	123	304	36	
Mean corpuscular volume, fL	102.7 ± 2.6	91.2 ± 5.1^†^	74.2 ± 4.6^†¥^	< 0.001
Age, years	56.1 ± 7.6	53.9 ± 7.1^†^	51.8 ± 6.2^**†**^	0.002
Male, n(%)	78(63.4)	191(62.8)	25(69.4)	0.738
Drinking, n(%)	24(19.5)	74(24.3)	13(36.1)	0.118
Smoking, n(%)	48(39.0)	107(35.2)	15(41.7)	0.618
Diabetes, n(%)	10(8.1)	39(12.8)	9(25.0) ^**†**^	0.026
Hypertension, n(%)	14(11.3)	36(11.8)	4(11.1)	0.985
Hemoglobin, g/L				
> 90	97(78.9)	230(75.7)	10(27.8)^†¥^	< 0.001
60–90	21(17.1)	63(20.7)	20(55.6)^†¥^	< 0.001
< 60	5(4.1)	11(3.6)	6(16.7)^†¥^	0.002
Total cholesterol, mmol/L	2.4 ± 0.7	2.7 ± 0.9^†^	2.7 ± 0.8	0.012
Systolic blood pressure, mmHg	117.9 ± 17.6	117.8 ± 15.2	113.8 ± 13.8	0.350
Diastolic blood pressure, mmHg	72.6 ± 11.7	73.4 ± 10.0	70.9 ± 8.6	0.354
Bilirubin, mg/dL	3.4 ± 3.4	2.6 ± 3.2^†^	1.8 ± 3.4^†^	0.011
Creatinine, mg/dL	0.8 ± 0.8	0.7 ± 0.4	0.7 ± 0.4	0.147
INR	1.5 ± 0.3	1.4 ± 0.4^†^	1.3 ± 0.1^†^	0.006
eGFR, mL/min/1.73m^2^	123.6 ± 54.5	126.9 ± 43.4	130.3 ± 39.1	0.686
Albumin	27.0 ± 4.7	29.1 ± 4.7^†^	31.7 ± 4.8^†¥^	< 0.001
AST	78.3 ± 147.3	84.2 ± 196.6	41.7 ± 39.5	0.394
ALT	45.3 ± 42.4	59.4 ± 114.3	29.3 ± 31.8	0.113
ALP	122.9 ± 55.5	106.4 ± 61.1^†^	85.2 ± 32.7^†^	0.001
MELD score	10.8 ± 6.6	8.0 ± 5.5^†^	6.3 ± 5.1^†^	< 0.001
Complications, n(%)				
UGB	6(4.9)	34(11.2)	5(13.9)	0.093
SBP	36(29.3)	75(24.7)^†^	3(8.3)^†^	0.037
HE	14(11.4)	22(7.2)	3(8.3)	0.377

Values are presented as mean ± standard deviation or numbers (percentage)

*INR* international normalized ratio, *eGFR* estimated glomerular filtration rate, *AST* aspartate aminotransferase, *ALT* alanine aminotransferase, *ALP* alkaline phosphatase, *MELD* model for end stage liver disease, *UGB* upper gastrointestinal bleeding, *SBP* spontaneous bacterial peritonitis, *HE* hepatic encephalopathy

*P* indicates the difference among the three groups. ^†^Indicates significance (*P* < 0.05) compared to macrocytic anemia; ^¥^Indicates significance (*P* < 0.05) compared to normocytic anemia

### Assessment of the association between MELD score and possible risk factors

We next assessed the correlation between the MELD score and possible risk factors using the univariate linear regression analyses (Table 2). Our results revealed a positive association between the MELD score and male, smoking and drinking. In addition, a negative association between the MELD score and the total cholesterol level was observed.

**Table 2:** Univariate and multivariate linear regression analysis for MELD score

Variable	Univariate		Multivariate	
	β (CI 95%)	*P* value	β (CI 95%)	*P* value
Age	0.05(−0.02,0.13)	0.160	0.07(0.01,0.15)	0.028
Male	2.25(1.15,3.36)	< 0.001	1.49(0.29,2.70)	0.015
Smoking	1.93(0.82,3.03)	< 0.001	0.21(−1.03,1.44)	0.742
Drinking	1.59(0.34,2.85)	0.013	0.73(−0.56,2.01)	0.269
Diabetes	0.53(− 1.11,2.16)	0.527		
Hypertension	0.15(−1.53,1.84)	0.857		
Hemoglobin, g/L				
> 90	Ref	–		
60–90	1.09(−0.21,2.39)	0.100		
< 60	−1.35(−3.90,1.20)	0.298		
Total cholesterol	−2.77(−3.37,-2.16)	< 0.001	−2.53(− 3.14,-1.93)	< 0.001
Systolic blood pressure	−0.01(− 0.04,0.03)	0.863		
Diastolic blood pressure	−0.01(− 0.06,0.05)	0.900		
Anemia classification				
Normocytic anemia	Ref	–	Ref	–
Macrocytic anemia	2.80(1.59,4.01)	< 0.001	1.94(0.81,3.07)	< 0.001
Microcytic anemia	−1.73(−3.72,0.27)	0.089	− 1.77(− 3.59,0.05)	0.057

*MELD* model for end stage liver disease, *β* estimated coefficient, *95% CI* 95% confidence interval

### Association between macrocytic anemia and MELD score

**Fig. 2:**
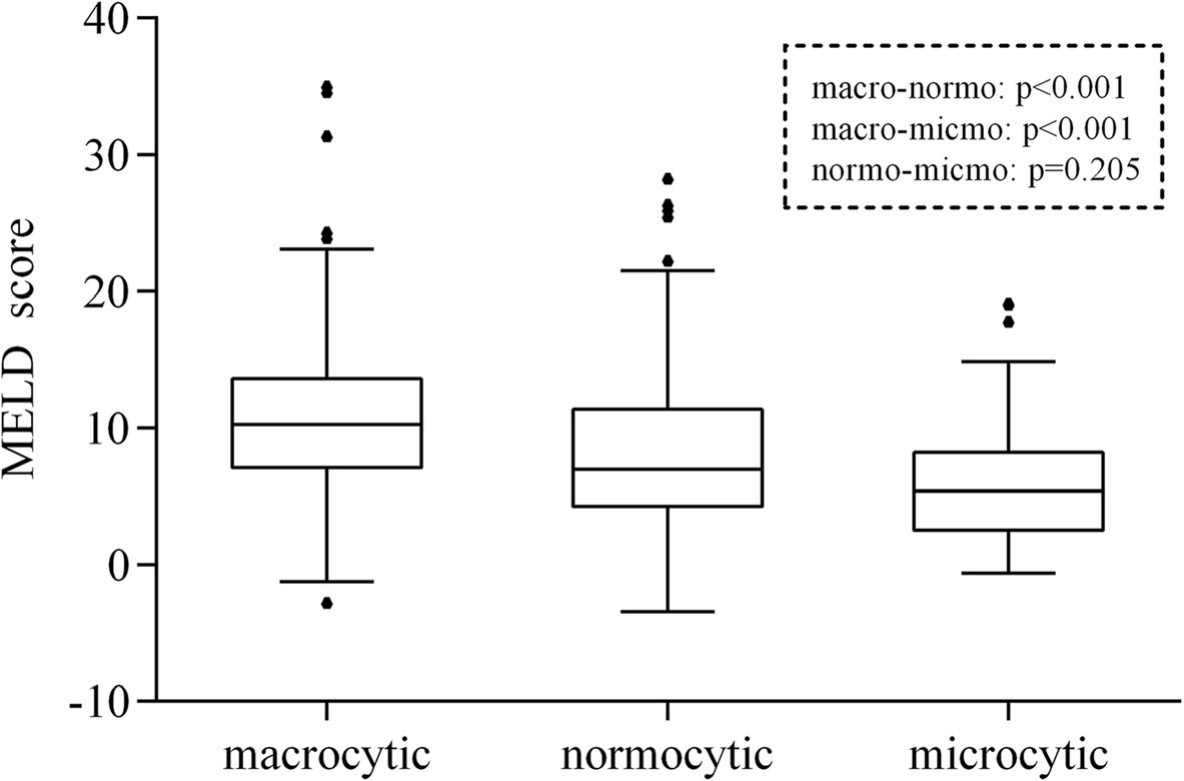
Anemia types and MELD scores. Patients with macrocytic anemia had evidently higher MELD scores than those with normocytic anemia (*P* < 0.001) or microcytic anemia (*P* < 0.001)

Patients in the macrocytic group had evidently higher MELD scores than patients in the other two groups (Fig. 2). In univariate regression analysis, we found that there was a significant association between macrocytic anemia and the MELD score (estimated coefficient [β] = 2.80, 95% confidence interval [CI]: 1.59–4.01, *P* value [P] < 0.001), using the normocytic group as the reference. Furthermore, the association remained robust (β = 1.94, CI: 0.81–3.07, *P* < 0.001) after adjusting for age, gender, smoking, drinking and total cholesterol in multivariate analysis (Table 2).

## Discussion

In this retrospective study, we demonstrated that macrocytic anemia, defined as anemia in which the RBCs are larger than their normal volume (100 fL), is associated with the severity of liver impairment in patients with HBV-related decompensated cirrhosis. This finding remains substantial even after adjusting for demographics and laboratory parameters, such as age, gender, smoking, drinking and total cholesterol.

An MCV level greater than 100 fL, which is also known as macrocytosis, may not always be associated with anemia. Moreover, it presents independently from anemia in most cases [10]. Nevertheless, we chose anemia as one of our inclusion criteria because 84.2% of the 1445 pre-screened patients have anemia. This result was consistent with the finding of another study, which reported that about 75% of patients with chronic liver disease have a diverse aetiology of anemia [25]. Furthermore, patients with cirrhosis may have anemia due to a lack of haematopoietic factors, shortened erythrocyte survival, reduced bone marrow function, or gastrointestinal bleeding. All these conditions indicate impaired liver function and a high risk of mortality. Therefore, patients without anemia were excluded from the data analysis to avoid potential bias in our present study.

The importance of macrocytic anemia or macrocytosis seems to be underestimated in the past. Only a few studies focused on its risk of adverse events or death [9–13]. Among these studies, Yoon et al. documented that the elevated MCV level was associated with increased liver cancer mortality in men [13]; this finding was consistent with the result of our study. A small-sample study also found a markedly higher MCV in patients with chronic liver failure than in healthy subjects [26]. These observations, though not directly, provided evidence for our conclusion that patients with HBV-related decompensated cirrhosis who have macrocytic anemia were more likely to present worse liver condition.

There are several potential pathological mechanisms that explain why macrocytic anemia is associated with the severity of liver impairment. First, patients with advanced liver damage are more likely to have vitamin B_12_ or folate deficiencies [27], which directly result in macrocytic anemia. Vitamin B_12_ and folate coenzymes are required for thymidylate and purine synthesis, thus, their deficiencies result in retarded DNA synthesis and eventually will develop into macrocytic anemia [28–30]. Second, macrocytic anemia in liver disease may be due to an increased deposition of cholesterol on the membranes of circulating RBCs [31, 32]. This deposition effectively increases the surface area of the erythrocyte. Third, hemolytic anemias are common in advanced liver failure. In this case, excessive destruction of RBCs and increased reticulocyte count can be observed. The immature erythrocytes are approximately 20% larger compared to the mature erythrocytes, which result in macrocytic anemia [25]. Moreover, erythrocyte morphology is affected by various factors in liver disease, such as causes, degree of liver damage, and drugs used. Complicated mechanisms, which allow the synchronized performance of their independent or collaborative functions, determine the shape of RBCs. Nevertheless, we firmly believe that there is a positive correlation between macrocytic anemia and the severity of liver impairment in patients with HBV-related decompensated cirrhosis.

In addition, we used the MELD score, which is a formula comprising creatinine, bilirubin, and INR values, to evaluate the severity of liver impairment and risk of death. In our study, patients with macrocytic anemia had higher levels of bilirubin and INR, but no significant difference was observed in creatinine levels and eGFR. Thus, macrocytic anemia might be unrelated to kidney damage in patients with HBV-related decompensated cirrhosis.

There were a few limitations in this study. First, we used the MELD score for evaluating the severity of liver impairment in patients with HBV-related decompensated cirrhosis. Although the MELD score could provide an accurate prediction of short-term mortality of patients with cirrhosis, a follow-up data might be better and more credible. Second, the analysis did not include data on serum vitamin B_12_, folate, reticulocyte count, drugs, and measures of haemolysis, which could contribute to better understand the mechanisms of macrocytic anemia in patients with cirrhosis.

## Conclusions

Macrocytic anemia was found to be associated with the severity of liver impairment and might be a predictor for short-term mortality in patients with HBV-related decompensated cirrhosis. However, a large-scale cohort study is recommended to confirm the present results and to elucidate the mechanisms underlying the observed correlations between macrocytic anemia and the severity of liver impairment in patients with HBV-related decompensated cirrhosis.

## Abbreviations

ALP: Alkaline phosphatase
ALT: Alanine aminotransferase
AST: Aspartate aminotransferase
BMI: Body mass index
eGFR: Estimated glomerular filtration rate
HBsAg: Hepatitis B surface antigen
HBV: Hepatitis B virus
HE: Hepatic encephalopathy
INR: International normalised ratio
MCV: Mean corpuscular volume
MDRD: Modification of diet in renal disease
MELD: Model for end stage liver disease
RBCs: Red blood cells
SBP: Spontaneous bacterial peritonitis
UGB: Upper gastrointestinal bleeding
